# Cinobufagin Enhances the Sensitivity of Cisplatin‐Resistant Lung Cancer Cells to Chemotherapy by Inhibiting the PI3K/AKT and MAPK/ERK Pathways

**DOI:** 10.1111/jcmm.70501

**Published:** 2025-03-26

**Authors:** Guangxin Zhang, Kun Zhang, Xin Li, Xiuwen Wang, Guangquan Li, Yicun Wang

**Affiliations:** ^1^ Department of Thoracic Surgery Second Hospital of Jilin University Chang Chun People's Republic of China; ^2^ Department of Medical Research Center Second Hospital of Jilin University Chang Chun People's Republic of China; ^3^ College of Life Science Jilin Normal University Si Ping People's Republic of China

**Keywords:** cinobufagin, cisplatin, drug resistance, lung cancer, traditional Chinese medicine

## Abstract

Lung cancer patients always develop serious chemotherapy resistance after long‐term use of cisplatin treatment. It has been demonstrated that the combination of cisplatin (DDP) with other chemotherapy drugs may significantly reduce drug resistance. Cinobufagin (CB) showed potent anti‐tumour effect against lung cancer. However, the relevance of CB and DDP resistance in lung cancer remains unclear. This article will study the effects of CB on reversing lung cell resistance. The apoptosis was rescued by flow cytometry analysis and TUNEL staining. The invasiveness was rescued by invasion assay. The mRNA and apoptosis‐related proteins were estimated by qRT‐PCR analysis and Western blot analysis, respectively. In vivo antitumor activities were investigated by subcutaneous xenograft assay. The present study firstly demonstrated that the sensitivity of DDP in DDP‐resistant A549 (A549/DDP) cells was enhanced when treated with CB. Moreover, CB combined with DDP weakened the proliferation and increased apoptosis of A549/DDP cells. In addition, the expression level of Bcl‐2 was increased, whereas Bax and caspase‐3 were activated when A549/DDP cells were treated with both drugs. After treatment with IGF1 or PMA and mixed drugs (CB + DDP), the expressions of P‐AKT, P‐PI3K, P‐MEK1/2 and P‐ERK1/2 were increased. Finally, the results of in vivo experiments showed that the combination of DDP and CB significantly reduced the growth of tumours derived from A549/DDP cells. The combination of CB and DDP can be considered an effective strategy to increase the sensitivity of DDP‐resistant lung cancer cells to DDP by inhibiting the PI3K/AKT and MAPK/ERK pathways.

## 1 Introduction

Up to now, lung cancer is still one of the malignant tumours that endanger human health, and its incidence is increasing year by year worldwide [1, 2, 3]. Due to the lack of effective early diagnosis methods and unobvious symptoms, most patients are already at an advanced stage when they are discovered and cannot be surgically removed. Chemotherapy has become the most commonly used method for the clinical treatment of lung cancer [3]. Cis‐dichlorodiamino platinum (cisplatin [DDP]) is a widely recognised first‐line chemotherapy agent for lung cancer [4]. However, prolonged use of cisplatin often leads to drug resistance, significantly reducing patient survival time and quality of life [5]. Therefore, developing effective strategies to overcome DDP resistance is crucial for improving lung cancer treatment outcomes.

Traditional Chinese medicine (TCM) has garnered significant attention in the field of drug development owing to its low toxicity and high efficacy, particularly in cancer treatment [6, 7]. Chan‐su is a traditional Chinese medicine recognised in several Asian countries. It is derived from the dry secretions and parotid glands of the Asian toad *Bufo gargarizans* [8]. Cinobufagin (CB), the primary active component of Chan‐su, is approved by the State Food and Drug Administration for treating liver and prostate cancer [9]. Substantial evidence has demonstrated that CB can inhibit the growth and progression of cancer [10, 11, 12]. For example, the cell cycle was arrested in the S phase and the apoptosis rate was improved when treated with CB in nasopharyngeal carcinoma cells [10]. Furthermore, Kim et al. found that CB suppressed melanoma cell growth by inhibiting Wnt/β‐catenin signalling via LEF1 inhibition [11]. It was discovered that CB inhibits the growth and tumourigenic potential of osteosarcoma cells by targeting the IL‐6‐OPN‐STAT3 signalling pathway [11]. Additionally, CB has been reported to inhibit the growth of non‐small cell lung cancer (NSCLC) cells and promote cancer apoptosis by inducing the AKT signalling pathway [12]. However, the mechanism of CB‐mediated resistance to lung cancer is poorly understood. To date, only one study has shown that CB non‐competitively reverses the chemoresistance of cancer cells by inhibiting the efflux function of P‐gp [13]. In this study, we demonstrate for the first time that CB can inhibit DDP resistance of A549 lung cancer cells both in vitro and in vivo through inhibiting the PI3K/AKT and MEK/ERK pathways.

## 2 Materials and Methods

### 2.1 Cell Culture

The human cancer cell lines A549 and A549/DDP were purchased from the cell bank of the Shanghai Chinese Academy of Sciences Type Culture Collection Committee, and the cells were placed in a constant‐temperature carbon dioxide cell incubator at 37°C and 5% CO_2_.

### 2.2 CCK‐8 Assay

The A549/DDP cells were seeded in a 96‐well plate at a density of 5 × 10^3^/well and exposed to CB, DDP, IGF1 (insulin‐like growth factor 1) and PMA (phorbol 12‐myristate 13‐acetate). Cells were subjected to DDP in group DDP, to CB in group CB, to CB combined with DDP in group CB + DDP, to IGF1 combined with mixed drugs (CB + DDP) in group IGF1 + CB + DDP and to PMA combined with mixed drugs (CB + DDP) in group PMA + CB + DDP. IGF1 (PMA) was added simultaneously with drug treatments for 30 min.

The A549 cells were seeded in a 96‐well plate at a density of 5 × 10^3^/well and were subjected to different concentrations of DDP (0, 0.5, 1, 2, 4, 8, 16 and 32 μM) for 24 h. The A549/DDP cells were subjected to different concentrations of CB (0, 0.25, 0.5, 1, 2, 4, 8 and 16 μM) or DDP (0, 5, 10, 20, 40, 80, 160 and 320 μM) for 24 h. In the combined group, (1) the A549/DDP cells were subjected to 0.1 μM CB and differnet concentration of DDP (0, 2, 4, 8, 16, 32, 64 and 128 μM), (2) the concentration of CB was 0.1 μM and that of DDP was 30 μM, the level of IGF1 was 100 ng/mL and PMA was 40 μM and that of mixed drugs (0.1 μM CB + 30 μM DDP). Then, the CCK‐8 solution was added to each well, and the plate was incubated for another 4 h and finally used the microplate reader (Thermo Fisher, US) to detect the absorbance at 450 nm and calculate the cell inhibition rate. In each group of the experiment, there were three replicates.

### 2.3 Flow Cytometry Analysis

The A549/DDP cells were cultured into 6‐well plates at a density of 5 × 10^5^/well and placed in a cell incubator for overnight culture, and the cells were divided into three or four groups and treated as above. Then digest cells with pancreatin (without EDTA) Cells were stained with Annexin V‐APC (abcam, US) for 10 min and 7‐AAD for 5 min (abcam, US) in the dark; finally, flow cytometry (Beckman, US) was used for collection and detection.

### 2.4 Invasion Assay

The A549/DDP cells were cultured in 6‐well plates at a density of 5 × 10^5^/well and placed in a cell incubator for overnight culture, allowing them to attach overnight in complete growth media. Cells were divided into three groups and treated as above. Before seeding the cells, the coated matrigel was spread in the upper chamber. The lower chamber was filled with medium containing 10% FBS. Cells were incubated for 24 h at 37°C and invaded through the matrigel. The cells adhering to the lower chamber were fixed with 4% paraformaldehyde for 30 min, stained with 0.5% crystal violet, and finally photographed with an inverted microscope.

### 2.5 qRT‐PCR Analysis

According to the manufacturer's instructions, TRIzol reagent (Thermo Fisher, US) was used to extract the total RNA in the cell. The total RNA was then reversely transcribed into cDNA according to the PrimeScript RT Reagent Kit instructions (Takara, Japan) and finally conducted the quantitative real‐time polymerase chain reaction (qRT‐PCR) analysis (Thermo Fisher, US). The PCR primer sequences are shown in Table 1.

**TABLE 1:** The PCR primer sequences used in the study.

Gene	Primer	Sequence
β‐Actin	Forward	5′‐AGCGAGCATCCCCCAAAGTT‐3′
	Reverse	5′‐GGGCACGAAGGCTCATCATT‐3′
MRP1	Forward	5′‐AGGTGGACCTGTTTCGTGAC‐3′
	Reverse	5′‐CCTGTGATCCACCAGAAGGT‐3′
Caspase‐3	Forward	5′‐ACTGGACTGTGGCATTGAGA‐3′
	Reverse	5′‐GCACAAAGCGACTGGATGAA‐3′
Bax	Forward	5′‐AAGAAGCTGAGCGAGTGTCT‐3′
	Reverse	5′‐GTTCTGATCAGTTCCGGCAC‐3′
Bcl2	Forward	5′‐GCCTTCTTTGAGTTCGGTGG‐3′
	Reverse	5′‐GAAATCAAACAGAGGCCGCA‐3′
MRP1	Forward	5′‐AGGTGGACCTGTTTCGTGAC‐3′
	Reverse	5′‐CCTGTGATCCACCAGAAGGT‐3′
AKT	Forward	5′‐ACACCAGGTATTTTGATGAGGAG‐3′
	Reverse	5′‐TCAGGCCGTGCCGCTGGCCGAGTAG‐3′
PI3K	Forward	5′‐AGCTGGTTTGGATCTTCGGA‐3′
	Reverse	5′‐CAGGTCATCCCCAGAGTTGT‐3′
MEK1	Forward	5′‐TGGCAATTTTTGAGTTGTTGGAT‐3′
	Reverse	5′‐TCTCTCTGCGGGGTTTTTTAT‐3′
MEK2	Forward	5′‐GAAGCGGCTGGAAGCCTTTCTC‐3′
	Reverse	5′‐GGGTCTGTGCTGGACTTTGGT‐3′
ERK1	Forward	5′‐TCAACACCACCTGCGACCTTAA‐3′
	Reverse	5′‐GTACCAGCGCGTAGCCACATA‐3′
ERK2	Forward	5′‐TTCCCAAATGCTGACTCCAAAGCT‐3′
	Reverse	5′‐TCACTCGGGTCGTAATACTGCTCC‐3′

The β‐actin was used as an internal reference, and the 2^−△△Ct^ method was used to analyse the relative expression levels of target genes.

### 2.6 Western Blot Analysis

The A549/DDP cells were cultured in 6‐well plates and placed in a cell incubator for overnight culture and were allowed to attach overnight in complete growth media. Cells were divided into three groups and treated as above. Total protein was extracted and denatured by boiling in water for 10 min, then protein concentration was measured by the BCA method. Take protein samples for gel electrophoresis, add primary antibodies (abcam, US), and incubate overnight at 4°C according to the product manual. After washing with PBS, membranes incubate with the secondary antibody at room temperature and detect the target protein in the gel imaging system after elution.

### 2.7 Subcutaneous Xenograft Assay

A total of 15 male BALB/c nude mice (6–8 weeks, 20–22 g) were housed under specific pathogen‐free (SPF) conditions. After digestion of A549/DDP cells with trypsin, a cell suspension was prepared with PBS and cultured into the groin of nude mice. Randomly grouped according to body weight, the experimental groups were divided intoa DMSO (1%) group, a monotherapy group (i.e., DDP), and a combination treatment group. After the tumour volume grew to 100–200 mm^3^, DDP and CB were injected intraperitoneally at a dose of 5 mg/kg and 3 mg/kg every 3 days for 51 days, respectively; the tumour size was measured with a vernier calliper, 51 days after the administration, the nude mice were sacrificed, and the tumour tissues were weighed and photographed.

### 2.8 Immunohistochemistry

The mouse tissue sections were deparaffinised with xylene (twice) for 15 min and hydrated with an alcohol series (100% twice, 90% twice, 80% once). After blocking the sections with 5% BSA, the sections were incubated with the primary antibody (abcam, US) overnight at 4°C. The next day, the sections were incubated with the secondary antibody at 37°C (abcam, US), and then treated with DAB chromogen and haematoxylin at room temperature, and finally observed with an inverted microscope.

### 2.9 TUNEL Assay

TUNEL staining was performed to determine apoptosis in each group following the manufacturer's instructions (abcam, US). The sections were deparaffinised with xylene and rehydrated using gradient ethanol. The permeable solution was then added for incubation at 37°C for 8 min. After rinsing with PBS, mixed TdT + dUTP was added to the treatment group, dUTP was added to the negative control group, and 100 μL DNase I was added to the positive control group and then incubated at 25°C for 10 min. The slides were then rinsed with PBS. The converter‐POD was added and incubated at 37°C for 30 min. After washing with PBS, the IDAB substrate was applied to the slides and reacted at 25°C for 10 min. The slides were then rinsed with PBS. Haematoxylin was added as a counterstain and sealed with neutral gum. The slides were observed using an optical microscope (Olympus, Japan) and the representative images were captured with a digital camera.

### 2.10 Statistics Analysis

All data were expressed as mean ± SEM. GraphPad Prism 8.0 software was used for statistical analysis. Student's *T*‐test was used to compare the two groups, and *p* < 0.05 was considered statistically significant.

## 3 Results

### 3.1 CB Enhanced the Inhibitory Effect of DDP Against A549/DDP Cells

**FIGURE 1:**
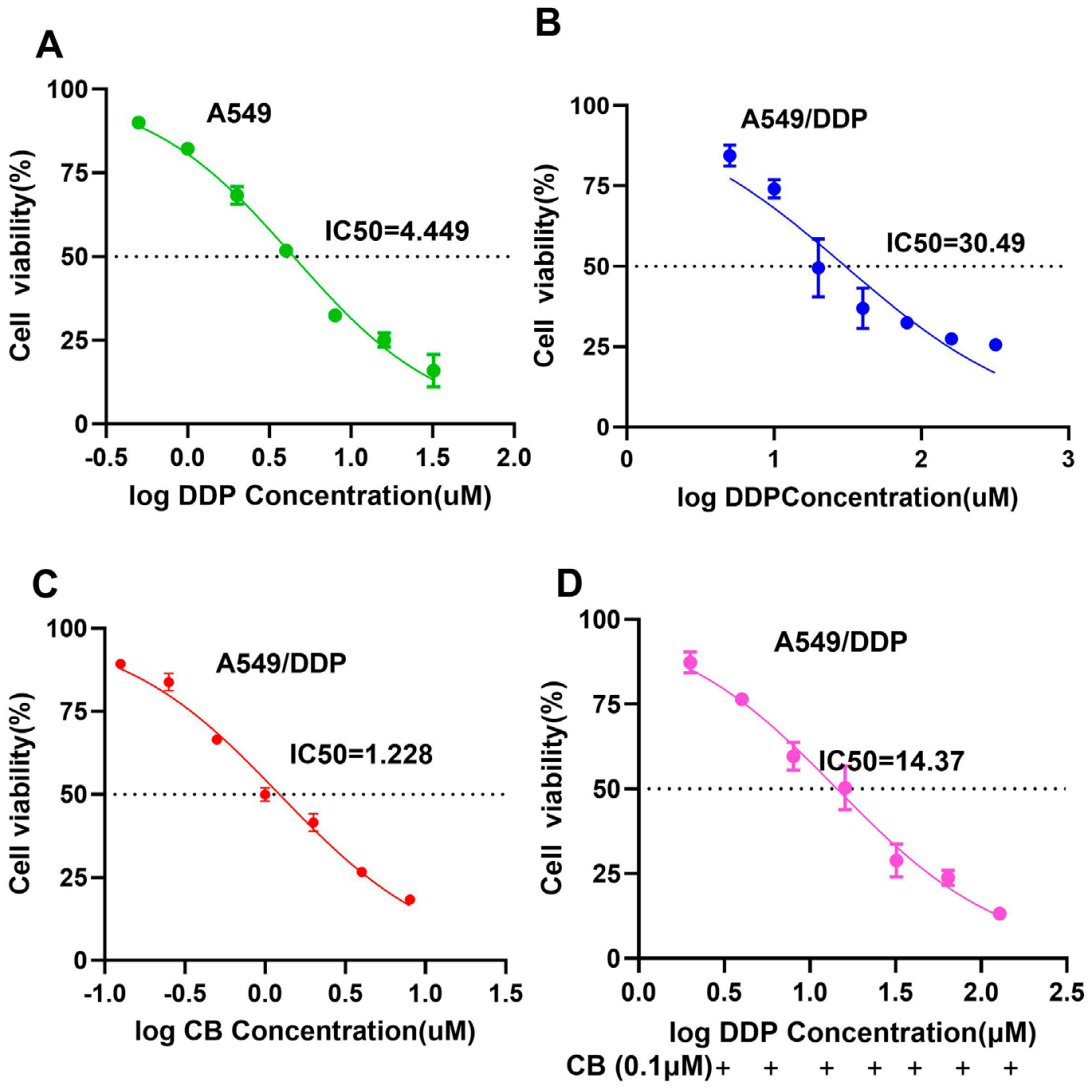
CB united with DDP can reduce the cell viability of DDP/A549 lung cancer cells. (A) The cell viability of A549 cells was detected with different concentrations of DDP for 24 h. Hill slope value = 0.9541 (B) The cell viability of A549/DDP cells was detected with different concentrations of CB, Hill slope value = 0.6813 (C) and DDP for 24 h, Hill slope value = 0.8654 (D). The cell viability of A549/DDP cells was treated with 0.1 μM CB combined with different concentrations of DDP for 24 h, Hill slope value = 0.8895. Compare 0.1% DMSO (v/v) group (*n* = 3).

Firstly, the cell viability of A549 and A549/DDP was examined by CCK8 assay in the study. It was found that DDP induced a significant loss of cell viability in the A549 cancer cells, whose IC_50_ reached 4.45 ± 0.35 μM (Figure 1A). Then, As shown in Figure 1B,C, both CB and DDP exhibited concentration‐dependent inhibitory effects on A549/DDP lung cancer cells, whose IC_50_ reached approximately 1.23 ± 0.13 μM and 30.49 ± 0.85 μM, respectively. Moreover, compared with the DDP group, the antigrowth effect of CB on the DDP/A549 cancer cells was significantly stronger, suggesting DDP/A549 cells were more sensitive to CB treatment. Furthermore, the combination inhibitory effect of CB and DDP was further studied. As shown in Figure 1D, the value of IC_50_ in the combination group exhibited an obviously lower value compared with the cells treated with DDP, which revealed that the combination of CB and DDP could significantly inhibit the viability of DDP/A549 cancer cells. According to the checkerboard method, it showed that different concentrations of CB could significantly improve the inhibition rate of DDP, and the combination of the two drugs had higher efficacy (Figure S1).

### 3.2 CB Combined With DDP Regulated Apoptosis and Invasion of DDP/A549 Cancer Cells

**FIGURE 2:**
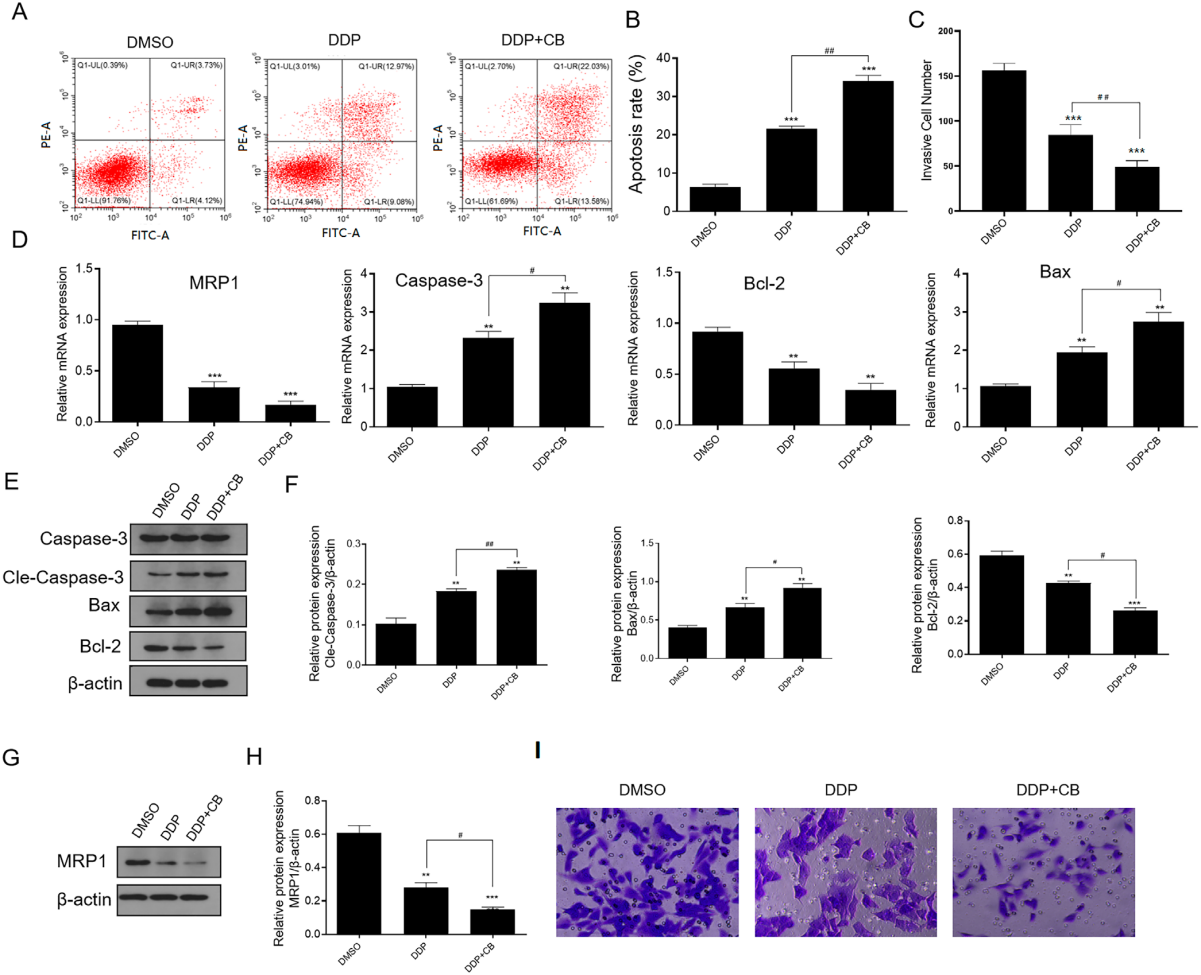
CB united with DDP increased apoptosis and reduced invasion. (A, B) The apoptosis rate of A549/DDP cellswase treated with 0.1 μM CB combined with 30 μM DDP for 24 h. (C) The mRNA expression of MRP1, caspase‐3, Bcl‐2 and Bax on A549/DDP cellswase treated with 0.1 μM CB combined with 30 μM DDP for 24 h. (D–G) The protein expression of caspase‐3, Bcl‐2, Bax and MRP1 on A549/DDP cellswase treated with 0.1 μM CB combined with 30 μM DDP for 48 h. (H) The invasion activity of A549/DDP cellswase treated with 0.1 μM CB combined with 30 μM DDP for 24 h. Compare 0.1% DMSO (v/v) group (*n* = 3), **p < 0.01, ***p < 0.001; Compare DDP group (*n* = 3), ^#^ *p* < 0.05, ^##^ *p* < 0.01.

Second, the synergistic effect of CB and DDP on the growth of DDP/A549 cancer cells related to cell apoptosis was further studied. As shown in Figure 2A,B, compared to the DMSO group, the apoptosis rates were increased in the DDP‐alone group and the combination group. Furthermore, compared to the DDP‐alone group, the combination effect of CB and DDP showed the highest apoptosis rate. It is well known that caspase‐3, Bcl‐2 and Bax have crucial roles in the execution of apoptosis. Therefore, we further studied the expression levels of apoptosis‐related proteins by qRT‐PCR and Western blot. As shown in Figure 2D, compared with the DMSO group, the cells treated with DDP displayed an obvious increase in mRNA expression of caspase‐3 and Bax, whereas the mRNA expression of Bcl‐2 decreased More importantly, these mRNA expressions of apoptosis‐related proteins were further regulated in combination group (Figure 2D). Subsequently, the results revealed that the expression level of apoptosis‐related proteins was a similar result in A549/DDP cancer cells (Figure 2E–H). Metastasis is the leading cause of death in lung cancer patients because of the aggressive ability of cancer cells [14]. After 24 h incubation, cells treated with DDP or CB in combination with DDP significantly decreased invasiveness, compared with the DMSO group (Figure 2C,I). These results demonstrated that CB in combination with DDP induced apoptosis and suppressed DDP/A549 cancer cell invasion. Furthermore, the expression of resistance‐related genes in A549/DDP cells was performed. As shown in Figure 2D,F–H, compared to the control group, the mRNA and protein expressions of the multi‐drug resistant protein 1 (MRP1) were downregulated in the DDP‐alone group and the CB in combination with DDP group, respectively.

### 3.3 CB Enhances the Inhibitory Effect of DDP on DDP/A549 Cancer Cells Through the PI3K/AKT Pathway and the MAPK/ERK Pathway

**FIGURE 3:**
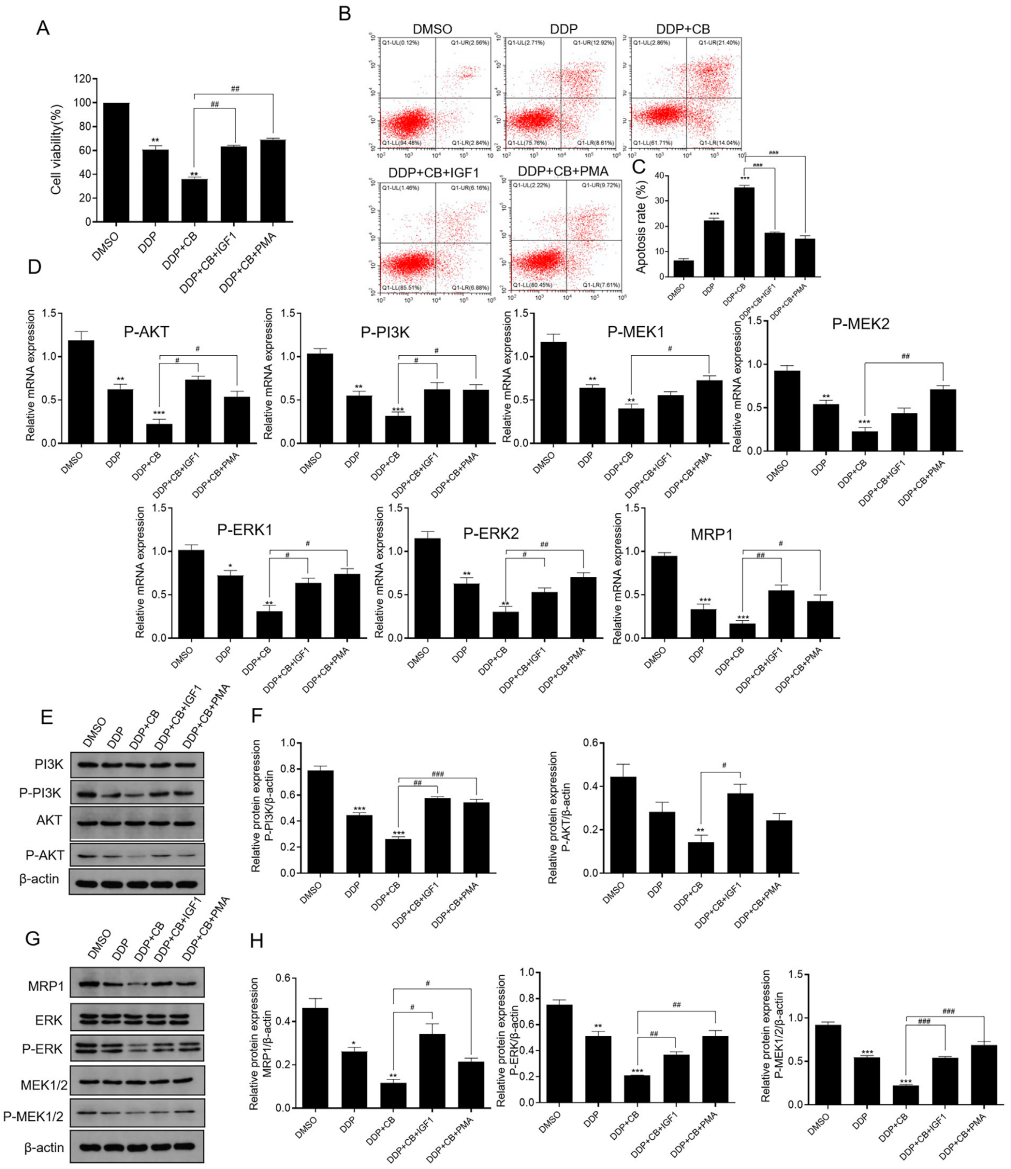
CB enhances the inhibitory effect of DDP on DDP/A549 cancer cells through the PI3K/AKT pathway and the MAPK/ERK pathway. (A) The cell viability of A549/DDP cells was detected with IGF1or PMA and mixed drugs (CB + DDP). (B) The apoptosis rate of A549/DDP cells was treated with IGF1or PMA and mixed drugs (CB + DDP). (C–H) The mRNA and protein expression of P‐AKT, P‐PI3K, P‐MEK1/2, P‐ERK1/2 and MRP1 in A549/DDP cells was treated with IGF1or PMA and mixed drugs (CB + DDP). Compare 0.1% DMSO (v/v) group (*n* = 3), *p < 0.05, **p < 0.01, ***p < 0.001; Compare DDP group, ^#^ *p* < 0.05, ^##^ *p* < 0.01, ^###^ *p* < 0.001.

Evidence has revealed that drug resistance is directly involved in the regulation of the intrinsic pathway or extrinsic pathway [15]. Recent studies revealed the key roles of the PI3K/AKT pathway and MAPK/ERK pathways in chemotherapy resistance [16, 17, 18, 19]. To explore whether CB affects A549/DDP growth by the PI3K/AKT and MAPK/ERK pathways, after treatment with IGF1 (activator of PI3K/AKT) or PMA (activator of MAPK/ERK) and mixed drugs (CB + DDP), as shown in Figure 3A–C, the cell viability and apoptosis rate of A549/DDP cells were increased and decreased compared with those in the single mixed drug treatment group, respectively. After pretreatment with IGF1, A549/DDP cells were incubated with the mixed drugs (CB + DDP). In these cells, the mRNA and protein expressions of P‐AKT and P‐PI3K were significantly increased compared with those in the single mixed drug treatment group, and the expression of P‐MEK1/2 and P‐ERK1/2 was also changed. Similarly, A549/DDP cells were incubated with PMA and mixed drugs successively; the expression levels of P‐MEK1/2 and P‐ERK1/2 were significantly increased compared with those in the mixed drug treatment group alone. More interestingly, A549/DDP cells were incubated with IGF1 or PMA, and the mRNA and protein expression of MRP1 were upregulated, suggesting that the PI3K/AKT and MAPK/ERK pathways may be related to MRP1. All data concluded that CB combined with DDP suppressed A549/DDP cell growth through the PI3K/AKT and MAPK/ERK pathways.

### 3.4 CB in Combination With DDP Inhibits the Growth of A549/DDP Cells In Vivo

**FIGURE 4:**
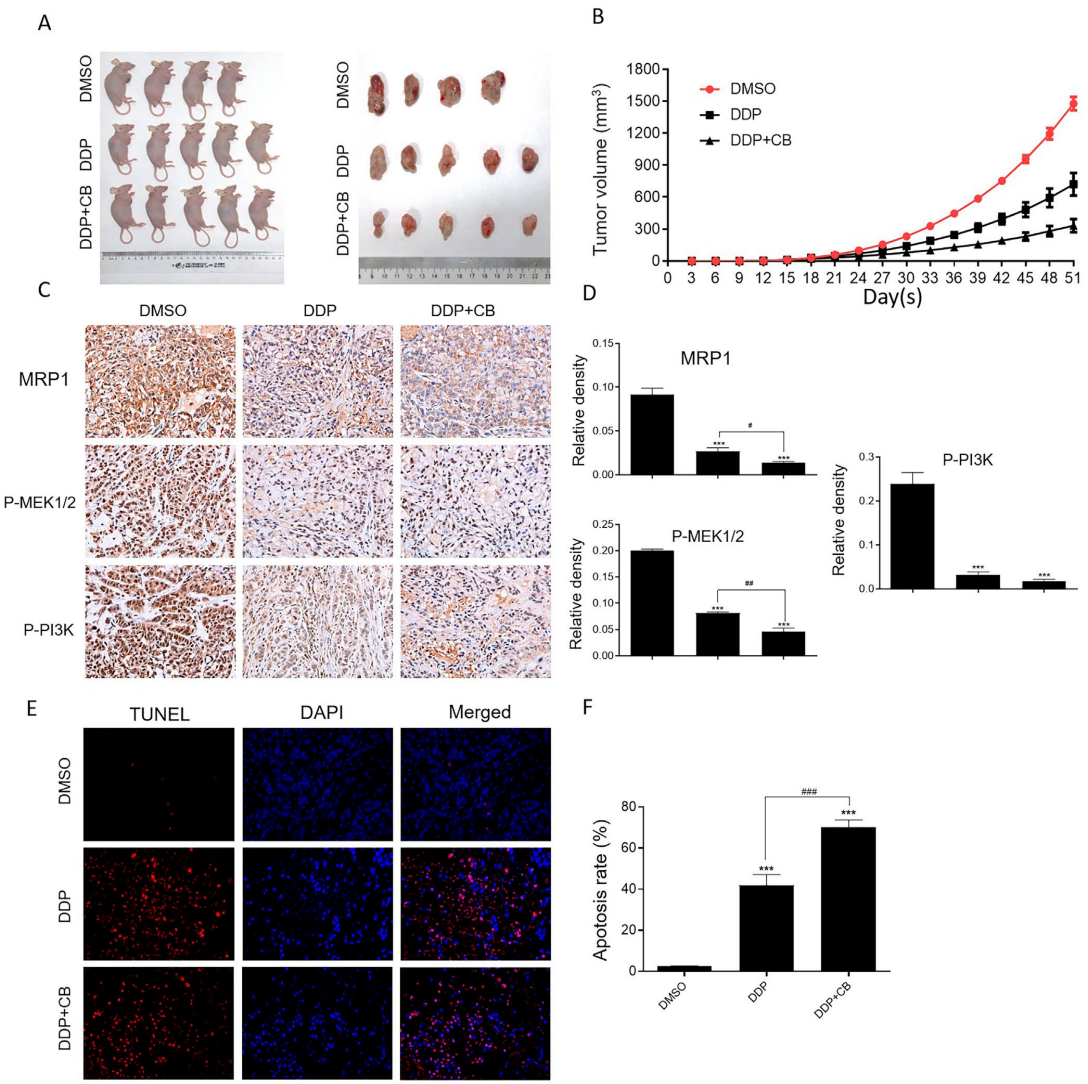
CB united with DDP inhibits the proliferation of A549/DDP cells in vivo. (A) The qualitative images and quantitative analysis (B) of tumour volumes after the treatment of A549/DDP tumour‐bearing mice with CB, DDP, and a combination of CB and DDP. (C, D) The qualitative (C) and quantitative (D) determination of MRP1, P‐MEK1/2 and P‐PI3K expression in tumour tissues by immunohistochemical staining. (E, F) The qualitative (E) and quantitative (F) determination of apoptosis in tumour tissues. Compare 1% DMSO (v/v), group ***p < 0.001; Compare DDP group, ^#^ *p* < 0.05; ^###^ *p* < 0.001.

We further investigated the synergistic effect of CB and DDP drug combination group in vivo. We established the subcutaneous ectopic tumour formation model by injecting A549/DDP cells into nude mice to evaluate the combined anti‐tumour effect of CB and DDP. The results showed that compared with the single drug groups, the lung tumour volume in the combination group was significantly lower, which suggested CB in combination with DDP could be an effective strategy for lung cancer treatment (Figure 4A,B). Moreover, immunohistochemical staining indicated that the densities of MRP1, P‐MEK1/2 and P‐PI3K were significantly more reduced in the combination group compared with those in single drug groups (Figure 4C,D). Furthermore, the results from the TUNEL assay also revealed the apoptosis rates in the combined drug group were significantly elevated (Figure 4E,F). All in all, these results indicate that DDP combined with CB had a good anti‐tumour effect in vitro.

## 4 Discussion and Conclusion

Despite significant advancements in therapeutic strategies, the prognosis for patients with advanced lung cancer remains poor, primarily due to the rapid development of chemotherapy resistance [1, 2]. Currently, DDP is a widely used chemotherapy agent for lung cancer patients. However, prolonged use of DDP often leads to drug resistance and adverse side effects, diminishing therapeutic efficacy and significantly compromising patient health [4, 5]. Studies have shown the 5‐year survival rate of lung cancer patients treated with DDP ranges from 4% to 17% [20]. Furthermore, studies have demonstrated that combining DDP with other drugs can significantly mitigate drug resistance, suggesting that combination therapy may be a potential treatment strategy to reduce single‐agent resistance [21, 22].

Previous studies have demonstrated that CB exhibits potent anti‐tumour activity against various cancers, including gastric cancer, lung cancer, breast cancer, osteosarcoma, pancreatic cancer and cancer‐related cachexia [23]. However, few studies have investigated the efficacy of CB against drug‐resistant cancers. In this study, we established a combined treatment strategy using CB and DDP to investigate their synergistic effects on A549/DDP lung cancer cells. The results revealed that the cell viability was significantly reduced in the combination group, compared with A549/DDP cells treated with DDP‐alone, suggesting that CB may enhance the sensitivity of A549/DDP cells to DDP. However, the underlying mechanism of this combination therapy remains unclear and requires further investigation. Further analysis demonstrated that the apoptosis rate of A549/DDP cells in the combination group was significantly increased, suggesting that CB enhances the apoptosis activity of DDP in A549/DDP cells. qRT‐PCR and Western blotting results indicated that the ability of CB to enhance DDP sensitivity may be primarily associated with the upregulation of proteins. In general, tumour cells have greater adaptability to the environment and robust independent survival capabilities, and the potential for unlimited invasion. Our results showed that A549/DDP cells treated with CB in combination with DDP significantly decreased invasiveness, indicating that the combination of CB and DDP suppresses lung cancer cell invasion. It has been reported in the literature that drugs can reverse chemoresistance of cancer cells that might be related to resistance‐related genes, including MRP1. qRT‐PCR analysis and Western blotting assays indicated that CB down‐regulated the expression of MRP1 in A549/DDP cells.

PI3K/AKT and MAPK/ERK pathways play critical roles in regulating cancer cell proliferation, migration, and metastasis. Moreover, numerous studies have demonstrated that DDP resistance is associated with dysregulation of cancer cell signalling pathways, particularly PI3K/AKT and MAPK/ERK pathways [24, 25]. Therefore, we investigated whether CB enhances DDP sensitivity by modulating the PI3K/AKT and MAPK/ERK signalling pathways. In our study, qRT‐PCR and Western blot assays were conducted to evaluate the effects of CB on the expression of the PI3K/AKT and MAPK/ERK pathway‐related proteins. Our results demonstrated that CB significantly inhibited the expression of AKT, PI3K, MEK1/2 and ERK1/2 at both mRNA and protein levels. However, when A549/DDP cells were treated with PMA or IGF1 in combination with CB and DDP, the expression levels of these proteins were upregulated. Furthermore, the results indicated that the inhibition of apoptosis rate was inhibited by adding PMA or IGF1, suggesting that CB sensitises DDP‐resistant A549/DDP cells to DDP through the PI3K/AKT and MAPK/ERK signalling pathways.

Based on the in vitro findings, we further investigated the anti‐lung cancer effect of CB combined with DDP on A549/DDP cells in vivo. The results demonstrated that CB increased the inhibitory effect of DDP on tumour growth, and the smallest tumour volume was observed in the combination group. Furthermore, the expression levels of MRP1, P‐MEK1/2 and P‐PI3K were significantly reduced in the combined group. In addition, we confirmed that the apoptosis rate was significantly increased following combined treatment with CB and DDP. Taken together, these findings indicate that CB significantly enhances the anti‐tumour efficacy of DDP.

In this study, the maximum tolerated dose (MTD) of CB was not determined experimentally, as prior research has established an MTD of at least 10 mg/kg in mouse models, with 3 mg/kg being a safe and effective dose in BALB/c nude mice [26, 27]. Although long‐term toxicity studies were not performed, existing evidence supports the safety and efficacy of combining CB with DDP. Studies have shown that this combination enhances antitumor effects while reducing systemic toxicity, such as myelosuppression, without significantly affecting body weight in animal models [28]. These findings suggest that CB combined with DDP is a promising therapeutic strategy with a favourable safety profile, warranting further investigation into its long‐term effects.

In conclusion, our findings demonstrate that CB enhances the sensitivity of chemotherapy‐resistant lung cancer cells to DDP by inhibiting the PI3K/AKT and MAPK/ERK pathways, providing a potential theoretical foundation for the treatment of DDP‐resistant lung cancer.

## Author Contributions

**Guangxin Zhang:** conceptualization (equal), methodology (lead), writing – original draft (supporting). **Kun Zhang:** data curation (supporting), formal analysis (supporting), software (supporting), validation (supporting). **Xin Li:** data curation (equal), formal analysis (lead). **Xiuwen Wang:** writing – review and editing (equal). **Guangquan Li:** software (lead). **Yicun Wang:** conceptualization (equal), funding acquisition (supporting), supervision (equal), writing – original draft (equal).

## Ethics Statement

The study was approved by the Ethics Committee of the General Hospital of the Second Hospital of Jilin University.

## Conflicts of Interest

The authors declare no conflicts of interest.

## Supporting information


**Figure S1.** CB in combination with DDP can reduce the cell viability of DDP/A549 lung cancer cells. The cell viability of A549 cells was detected with different concentrations of DDP and CB for 24 h by checkerboard. (A) The cell viability of A549/DDP cells was significantly induced with different concentrations of CB and Fixed concentration DDP for 24 h. (B) The cell viability of A549/DDP cells was significantly induced treated with fixed concentration CB combined with different concentrations of DDP for 24 h. Compare DMSO group, (*n* = 2) **p < 0.01.

## Data Availability

All data generated or analysed during this study are included in this published article and its Supporting Information. Additional datasets are available from the corresponding author upon reasonable request.
