# Supplementary material for: Cinobufagin Enhances the Sensitivity of Cisplatin‐Resistant Lung Cancer Cells to Chemotherapy by Inhibiting the PI3K/AKT and MAPK/ERK Pathways
Source: J Cell Mol Med. 2025 Mar 26;29(6):e70501. doi: 10.1111/jcmm.70501 (PMC11937849; doi:10.1111/jcmm.70501)

**Supplementary Material**

**Cinobufagin enhances the sensitivity of cisplatin-resistant lung cancer cells to chemotherapy by** **inhibiting the PI3K/AKT and MAPK/ERK pathways**

Guangxin Zhang^a*^, Kun Zhang^b^, Xin Li^b^, Guangquan Li^b^, Yicun Wang^b*^

1. Department of Thoracic Surgery, Second Hospital of Jilin University, Chang Chun, 130041, PR China.
2. Department of Medical Research Center, Second Hospital of Jilin University, Chang Chun, 130041, PR China.

Correspondence to: Yicun Wang, Second Hospital of Jilin University, Changchun, PR China. Email: [wangyicun@jlu.edu.cn](mailto:tongti@jlu.edu.cn)

1. Development of CB Solutions for Cell and Animal Experiments

CB is a major active component of the traditional Chinese medicine Chan-su. It has limited solubility in water due to its hydrophobic natur1. We dissolve CB in DMSO to prepare a high concentration stock solution. All solutions are ready-to-use. In cell experiments, to prepare an 8 mM stock solution, we dissolve 1 mg of CB in 0. 2825 mL of DMSO, to ensure that the final DMSO concentration in the cell culture medium does not exceed 0.1% (v/v). For an 8 μM final concentration in 1 mL of medium: Add 10 μL of the 8 mM stock solution to 9.99 mL of cell culture medium. This will result in a final DMSO concentration of 0.1% (v/v), which is within the safe limit. After adding the DMSO stock solution to the cell culture medium. In animal experiments, the preparation method of stock solution: 0.3 mg drug dissolved in 10 μL DMSO. Preparation method of working solution in animal experiment: take 10 μL DMSO stock solution, add 20 μLPEG300 Mix well until clarified, then add 5 μL Tween 80, mix well until clarified, and then add again 965 μL Saline. Ultrasonic heating is used to help the solute dissolve during the preparation of the solution (frequency 30 kHz).

2.To evaluate the potential toxicity of the combination of CB and DDP, we used the CCK-8 assay to measure cell viability in A549/DDP cells treated with either individual drugs or the drug combination.

**Method:**

1). Cell Culture: A549/DDP cells were used for the experiments. Cells were maintained in RPMI-1640 medium supplemented with 10% FBS at 37°C with 5% CO_2_

2). Drug Preparation: Stock solution was prepared in DMSO and diluted to the desired concentrations in culture medium.

3). CCK-8 Assay

The A549/DDP cells were seeded in a 96-well plate at a density of 5×103/well and subjected to different concentrations of CB (0, 0.6, 1.2, 2.4 μM) or DDP (0, 15,30, and60 μM) for 24 h, using the checkerboard method to set the concentration. Control: Cells treated with vehicle (0.1% DMSO). After incubation, 10 μL of CCK-8 solution was added to each well. Plates were incubated for an additional 4 hours at 37°C.The absorbance was measured at 450 nm using a microplate reader (Thermo Fisher).

**Result:**

According to the checkerboard method, the cell viability rate was calculated, and the results of CCK8 experiment showed that IC50, 1/2 concentration, and 2-fold concentration of CB could significantly improve the inhibition rate of DDP , and the combination of the two drugs had higher efficacy.

Figure S1.CB in combination with DDP can reduce the cell viability of DDP/A549 lung cancer cells. The cell viability of A549 cells was detected with different concentrations of DDP and CB for 24h by checkerboard. (A) The cell viability of A549/DDP cells was significantly induced with different concentrations of CB and Fixed concentration DDP for 24h. (B) The cell viability of A549/DDP cells was significantly induced treated with fixed concentration CB combined with different concentrations of DDP for 24 h. Compare DMSO group, （n=2） ** *p < 0.01*.


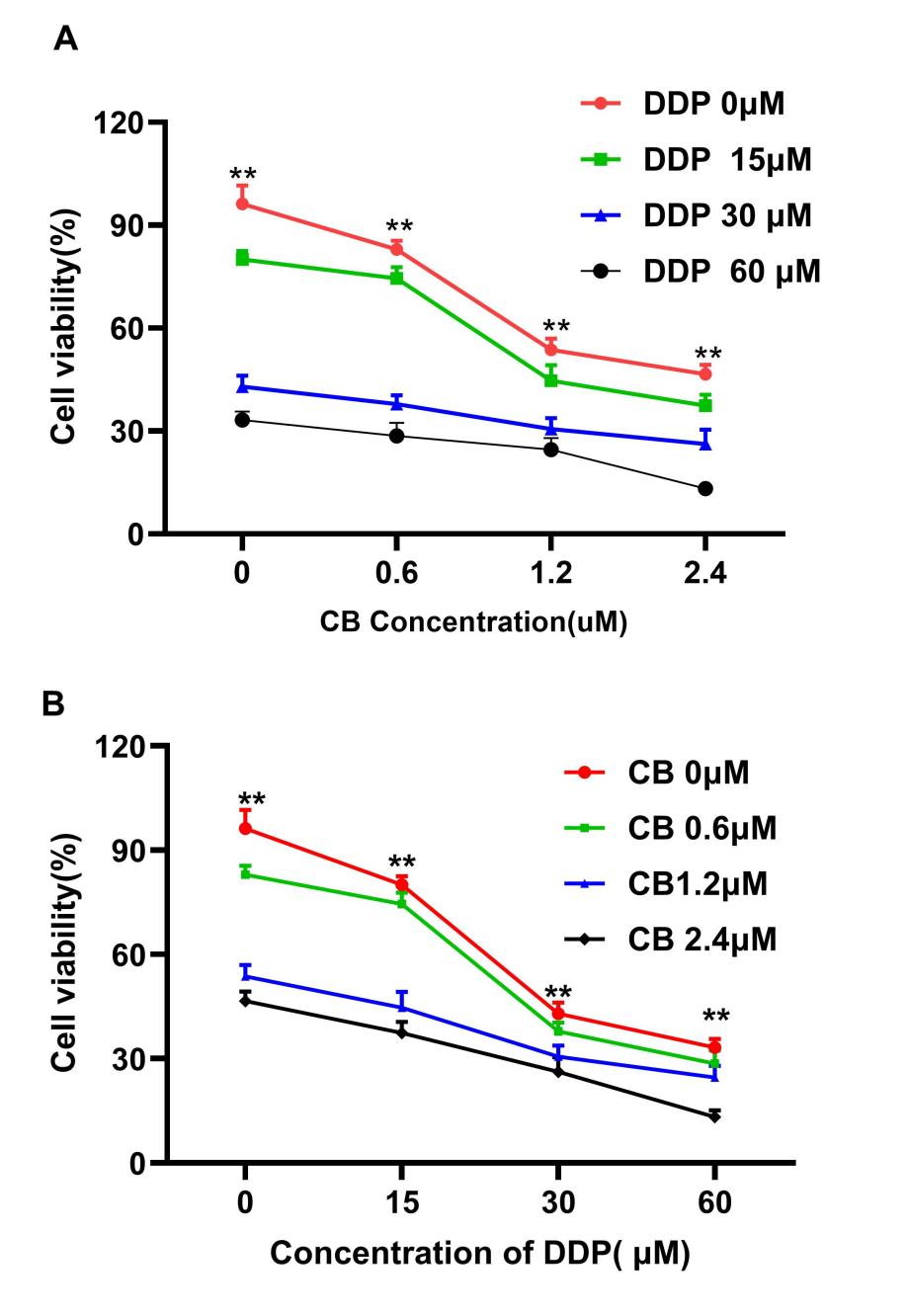

Supplement: Supplementary file 1 — Figure S1. CB in combination with DDP can reduce the cell viability of DDP/A549 lung cancer cells. The cell viability of A549 cells was detected with different concentrations of DDP and CB for 24 h by checkerboard. (A) The cell viability of A549/DDP cells was significantly induced with different concentrations of CB and Fixed concentration DDP for 24 h. (B) The cell viability of A549/DDP cells was significantly induced treated with fixed concentration CB combined with different concentrations of DDP for 24 h. Compare DMSO group, (n = 2) **p < 0.01. [file JCMM-29-e70501-s001.docx]
